# Epstein–Barr virus-related dacryocystitis: a case report 

**DOI:** 10.1186/s13256-022-03646-7

**Published:** 2022-11-19

**Authors:** J. Sternberg, S. Lambiel, H. Cao Van, H. Massa, B. N. Landis

**Affiliations:** 1 Department of Pediatrics, Geneva University Hospital, Geneva, Switzerland; 2 Department of Clinical Neurosciences, Oto-Rhino-Laryngology and Head and Neck Surgery, Geneva University Hospital, Geneva, Switzerland; 3 Department of Clinical Neurosciences, Ophthalmology Unit, Geneva University Hospital, Geneva, Switzerland

**Keywords:** Case report, Dacryocystitis, Epstein–Barr virus, Pediatrics

## Abstract

**Background and objective:**

Acute dacryocystitis is an atypical and rare manifestation of pediatric mononucleosis still widely underdiagnosed in clinical practice. We report this rare condition and describe challenges in its diagnosis and treatment on the basis of a presented case.

**Case presentation:**

A 6-year-old Caucasian girl without any ophthalmic history was admitted for right preseptal cellulitis requiring intravenous antibiotic therapy. During hospitalization, she developed a fluctuating lump in the nasolacrimal region which resembled an abscess, both clinically and radiologically. There was no spontaneous purulent discharge. Serology was positive for acute mononucleosis and Epstein–Barr virus-related dacryocystitis was diagnosed. Following multidisciplinary discussion, she was treated conservatively with digital lacrimal sac massages and intravenous antibiotic therapy with an excellent outcome.

**Discussion:**

This rare form of Epstein–Barr virus is poorly documented in the literature, and thus barely known. As initial symptoms are nonspecific (rhinitis, fever, eyelid edema and erythema lack of purulent discharge, and moderate bilateral cervical lymphadenopathy), diagnosis is often difficult. Nevertheless, differentiating between dacryocystitis and abscess is crucial to select the appropriate treatment and avoid unnecessary, potentially harmful surgery. Conservative management of dacryocystitis appears to be the gold standard of treatment.

**Conclusion:**

Acute dacryocystitis in children free of ophthalmic history should raise suspicion of primary Epstein–Barr virus infection. With conservative treatment, prognosis appears to be excellent; therefore, surgery should be avoided as much as possible.

## Introduction

In the adult population, the seroprevalence of Epstein–Barr virus (EBV) is 90–95%, with most primary infections occurring during childhood and adolescence [1–4]. While the majority of infections are subclinical, some present the typical triad of fever, lymphadenopathy, and pharyngitis. Only a few patients develop hepato- or splenomegaly [3–5]. While these are the classical manifestations, many others manifestations have been described [2, 6]. Lacking awareness of these less common clinical presentations may result in diagnostic errors or delay and suboptimal treatment.

In the pediatric population, acute dacryocystitis by obstruction of the nasolacrimal duct (due to nasal mucosal hypertrophy) is an atypical presentation of mononucleosis [2, 5, 7–9]. This case report aims to familiarize physicians with this uncommon presentation. Accurate diagnosis is necessary to choose the appropriate treatment.

## Case report/clinical case

We report the case of a 6-year-old Caucasian girl hospitalized for right orbital preseptal cellulitis, requiring intravenous antibiotic therapy. Medical history included adenotonsillectomy at the age of 2 years and a second adenoid resection at the age of 5 years. She had no ophthalmological history.

Upon emergency room admission, she presented erythematous, sensitive, and edematous eyelids that had developed after 5 days of fever (up to 39.5 °C) and 2 weeks of unilateral clear right eye tearing. A suspicion of bacterial conjunctivitis had been treated with topical antibiotics (ofloxacin) but with no improvement.

She had also reported chronic nasal obstruction, noisy breathing, and rhinolalia over the past 4–6 weeks, suggesting adenoid hypertrophia relapse, as well as purulent anterior rhinorrhea for the last 7 days.

**Fig. 1:**
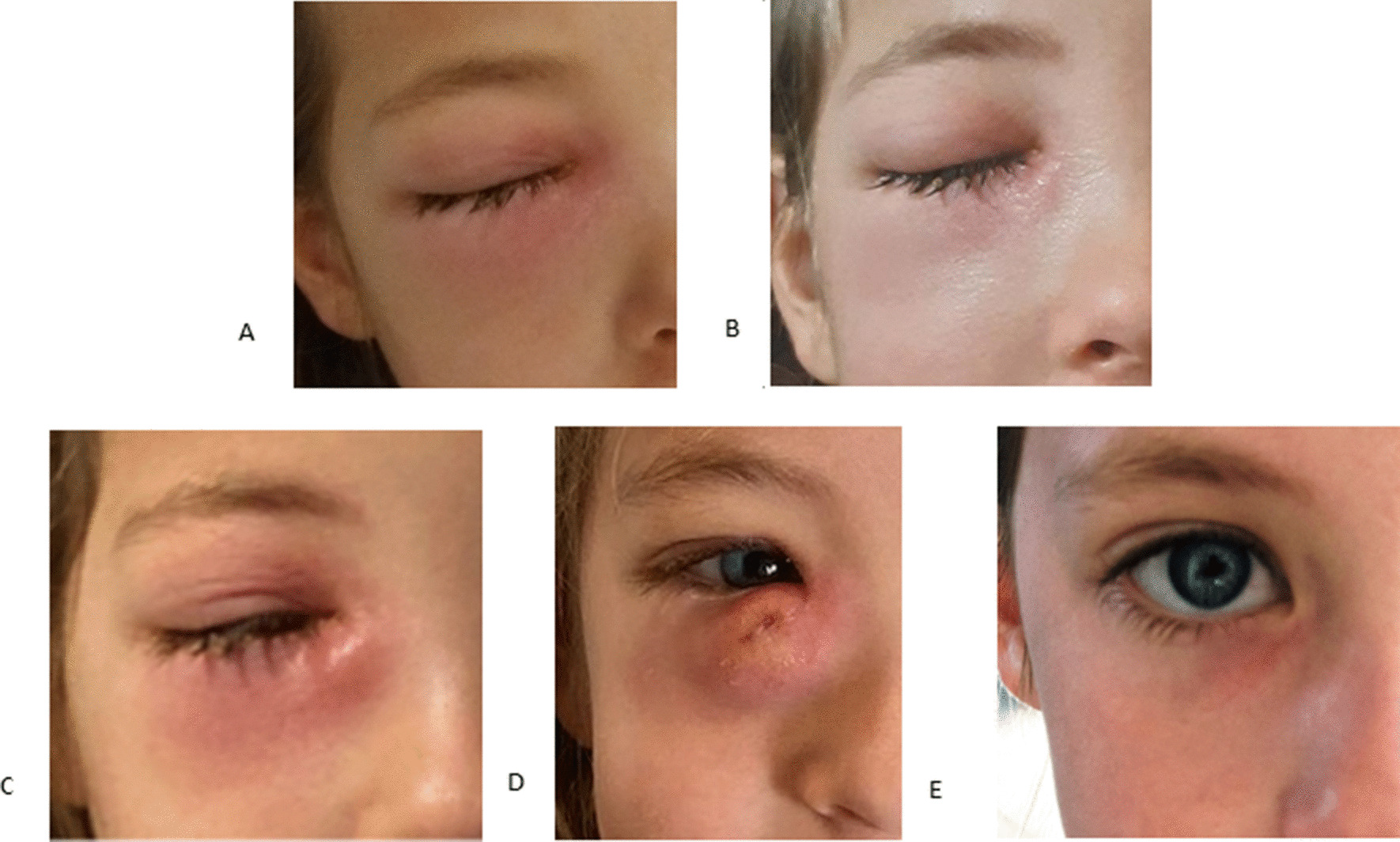
Pictures (**A**) at admission, (**B**) on day 2 (**C**), on day 3 (**D**), on day 8 of follow-up (**E**), and 3 months later

Examination showed a right periorbital tumescence predominant on the upper eyelid (Fig. 1A), with no ocular discharge. Nasal mucosae were swollen and bilateral neck lymphadenopathy was present, predominantly on the right side. Detailed neurological examination was normal. Ocular movements were within normal range and there was no orbital protrusion. Snellen uncorrected distance visual acuity was 20/20 in both eyes, and there was no relative afferent pupillary defect. The anterior segment on the right side showed an “S”-shaped palpebral tumefaction with an erythema of the skin overlying the lacrimal sac. Also, palpation of the nasal canthus was painful. The posterior segment was calm (the vitreous humor and the papilla were clear, the macula had no special features, the blood vessels were regular, and the retina was flat). There was no papilledema and no signs of hemorrhages. In addition, abdominal examination did not reveal organomegaly and the back of the throat was not inflammatory.

**Fig. 2:**
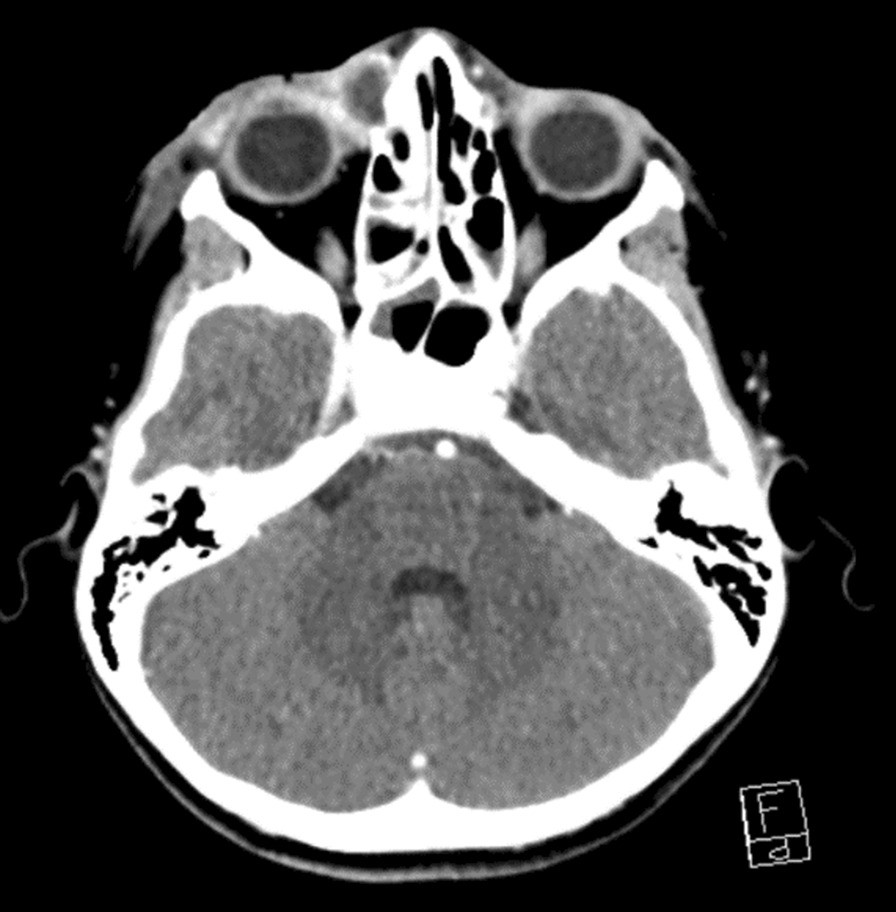
CT scan showing a right preseptal infiltration attributed to cellulitis with an ipsilateral well-delimited collection measuring 11 × 18 × 13 mm. Multiple bilateral cervical lymphadenopathies are also present and concordant with acute EBV infection

Blood analysis revealed a minor inflammatory syndrome (normal white blood cell count, left shift, elevated C-reactive protein at 22 mg/l and normal procalcitonin). Owing to the suspicion of a complication in the paranasal sinus, a cerebral computed tomography (CT) scan was performed (Fig. 2). It showed right preseptal infiltration attributed to cellulitis, as well as an ipsilateral well-delimited collection measuring 11 × 18 × 13 mm, with enhanced borders suggesting a periorbital abscess. The rest of the CT scan was reassuring and did not reveal any signs of acute sinusitis or intracranial complications.

The patient was treated with intravenous co-amoxiclav 100 mg/kg/day in three doses, as well as topical ocular antibiotics (one drop three times per day of neomycine 3.5 mg/ml and polymyxine B sulfate 6000 U/ml). Nevertheless, clinical status progressively deteriorated (Fig. 1B and C). She developed a fluctuating lump in the nasolacrimal region and daily fever persisted up to 96 hours after admission. Upon consensus with ophthalmologist and otorhinolaryngologist specialists, oral systemic corticosteroids were introduced to reduce inflammation. No spontaneous purulent discharge was observed but the lacrimal sac distension became increasingly compatible with a cutaneous abscess (Fig. 1D). Blood cultures and bacteriological swabs of the eye discharge as well as methicillin-resistant *Staphylococcus aureus* (MRSA) screening were all negative. Nonetheless, in light of this clinical deterioration, surgical drainage and nasolacrimal pathway probing were considered.

At this point, EBV serology came back positive with highly elevated antibodies (immunoglobulines G and M), compatible with an acute infection.

We concluded that EBV primary infection resulted in acute right dacryocystitis, complicated by preseptal ipsilateral cellulitis and adenoid hypertrophy. The multiple bilateral cervical adenopathies were also compatible with this infection.

The excellent general condition of our patient, as well as the slow but favorable evolution motivated the decision to maintain conservative treatment. Digital ocular massages were performed but they allowed only minimal drainage in the internal canthus of the eye. Intranasal corticoids were introduced for the chronic nasal obstruction and adenoid hypertrophy.

On day 8 of the antibiotic regimen, the patient developed a morbilliform rash, justifying the switch from oral co-amoxiclav 80 mg/kg/day in three doses to oral clindamycin 30 mg/kg/day in four doses for a further 13 days (total treatment duration of 21 days). Evolution was slow but gradual improvements were noted. Surgical drainage was avoided and our patient was discharged from the hospital after 1 week.

Follow-up at 1 month showed no sequelae, except for discrete skin redness under the right eye (Fig. 1E). Periorbital eye swelling and nasal obstruction had completely resolved and tears were evacuated properly by the nasolacrimal duct.

## Discussion

Once inoculated, EBV replicates in the naso- and oropharyngeal epithelium, and then spreads rapidly to neighboring lymphoid tissues such as tonsils, adenoids, and nasal mucosae [3]. In children, nasolacrimal anatomy is small and such an inflammation can induce nasal mucosal congestion, and therefore an acute obstruction of the nasolacrimal duct. Thus, tears are not properly drained through the Hasner valve and have a tendency to accumulate in the lacrimal sac [1, 2].

Stagnation of secretions, whatever the cause, can result in secondary infections with respiratory pathogens (most frequently *Streptococcus pneumoniae*, *Haemophilus influenza*, or *Staphylococcus aureus*) [2, 7, 9]*.* Clinically, pain and tenderness increase and the infection may extend and diffuse to adjacent tissues. For this reason, antibiotic therapy (systemic and topical) seems justified and is recommended as first-line therapy for acute dacryocystitis [10, 11].

Antiinflammatory drugs, such as ibuprofen and corticoids can be used in addition to decrease tissue swelling and favor natural tear drainage and evacuation [8].

It is important to note that acute lacrimal retention can also be caused by dacryoliths, mucus plugs, blood clots, foreign bodies, mucosal stenosis, or bacterial infection with empyema [8]. The real challenge lies in recognizing EBV as the cause of dacryocystitis and differentiating it from acute lacrimal retention of another origin or an abscess. It is essential to perform an accurate diagnosis to choose the appropriate treatment. The most common origin of periorbital cellulitis in the pediatric emergency setting is ethmoiditis or local eyelid abscesses that have very similar clinical presentations with early dacryocystitis [12]. They should also be considered in the differential diagnosis and taken into consideration when treatment strategy is decided. CT imaging can help to identify the cause but can also create confusion by revealing abscess-like lesions that correspond to an inflamed and distended lacrimal sac.

In this case scenario, given the typical localization visible since day 2 (Fig. 1b), a regurgitation on the pressure over lacrimal sac (ROPLAS) test could have been done. This could have helped us to make the diagnostic of acute dacryocystitis and maybe spared the patient a scan. Nevertheless, palpation was very painful and therefore difficult, and the nasolacrimal duct was very congested, almost obstructed, so a regurgitation would probably not have been obtained properly.

In contrast to abscesses due to eyelid infections or sinusitis that often require surgery [10, 12], invasive therapy for pediatric dacryocystitis is controversial. It is reserved for chronic, persistent dacryocystitis, or children presenting with congenital nasolacrimal duct obstruction, which is not the case in EBV-related cases [10, 11]. Percutaneous puncture and drainage is suggested if dacryocystitis is complicated with an adjacent abscess. Nasolacrimal probing, intubation, or stenting are also options but usually discouraged in the acute phase owing to the risk of secondary stenosis [13]. Other invasive therapies such as balloon dacryoplasty, percutaneous dacryocystorhinostomy, or endonasal dacryocystorhinostomy exist but are mostly reserved for adults [13–15].

In the case of acute dacryocystitis associated with mononucleosis, there are no clear treatment guidelines. The main goal is to unclog the lacrimal sac and prevent a potential infection. Conservative management (digital massages, intravenous antibiotic therapy, topical antibiotics with or without systemic corticoids) seems to be the consensus approach among specialists [1, 2, 5, 7, 8, 13]. It is of note that antibiotic therapy with aminopenicillins is not indicated if EBV infection is clearly documented because, in 70–90% of these cases, a rash can appear. Pathogenesis of this secondary cutaneous eruption is not fully understood and the causality with antibiotic therapy is debated [3, 5]. Literature is scarce but recommends avoiding surgery in the absence of complications such as preseptal cellulitis, ethmoiditis, cavernous sinus thrombosis, meningitis, and abscess [1, 2, 5, 7, 8]. Invasive treatment seems to be inadequate owing to the risk of cutaneous fistula or postoperative stenosis [16]. In this case, it is important to be aware of the possible ongoing viral fever in a patient undergoing antibiotic therapy, which could encourage unnecessary invasive treatment.

Corticosteroid therapy has only been used in a small number of cases of dacryocystitis, but its effectiveness remains to be proven [8].

## Conclusion

In the pediatric population, acute dacryocystitis is very rare. If no history of congenital obstruction of the nasolacrimal duct is present, one should always suspect a primary EBV infection and conduct necessary screening tests [2].

Despite the small number of cases described in the literature (less than 20), all report that uncomplicated acute EBV-induced dacryocystitis has a favorable prognosis with conservative treatment. Once the infection is managed, symptoms tend to disappear spontaneously without any residual nasolacrimal dysfunction [2, 5, 7–9]. Lacrimal irrigation and fluorescein permeability tests have sometimes been performed to confirm this [9].

While a secondary bacterial infection should always be considered, swelling is most often caused by distention of the lacrimal sac filled with tears resulting from obstruction and not from a coexisting abscess. Therefore, invasive treatment should be avoided.

## Data Availability

Not applicable.

## Abbreviations

EBV: Epstein–Barr virus
CT: Computed or computerized tomography
